# Metabolic and functional reprogramming of myeloid-derived suppressor cells and their therapeutic control in glioblastoma

**DOI:** 10.15698/cst2019.02.176

**Published:** 2019-01-23

**Authors:** Woong-Jai Won, Jessy S. Deshane, Jianmei W. Leavenworth, Claudia R. Oliva, Corinne E. Griguer

**Affiliations:** 1 Department of Neurology, University of Alabama at Birmingham, Birmingham, AL 35294, USA.; 2 Department of Medicine, University of Alabama at Birmingham, Birmingham, AL 35294, USA.; 3 Department of Neurosurgery, University of Alabama at Birmingham, Birmingham, AL 35294, USA.; 4 Free Radical and Radiation Biology Program, The University of Iowa, Iowa City, IA 52242, USA.

**Keywords:** glioblastoma, MDSCs, metabolic reprogramming, temozolomide (TMZ), immunotherapy

## Abstract

Glioblastoma, also known as glioblastoma multi-forme, is the most common and deadliest form of high-grade malignant brain tumors with limited available treatments. Within the glioblastoma tumor microenvironment (TME), tumor cells, stromal cells, and infiltrating immune cells continuously interact and exchange signals through various secreted factors including cytokines, chemokines, growth factors, and metabolites. Simultaneously, they dynamically reprogram their metabolism according to environmental energy demands such as hypoxia and neo-vascularization. Such metabolic re-programming can determine fates and functions of tumor cells as well as immune cells. Ultimately, glioma cells in the TME transform immune cells to suppress anti-tumor immune cells such as T, natural killer (NK) cells, and dendritic cells (DC), and evade immune surveillance, and even to promote angiogenesis and tumor metastasis. Glioma-associated microglia/macrophages (GAMM) and myeloid-derived suppressor cells (MDSC) are most abundantly recruited and expanded myeloid lineage cells in glioblastoma TME and mainly lead to immunosuppression. In this review, of myeloid cells we will focus on MDSC as an important driver to induce immunosuppression in glioblastoma. Here, we review current literature on immunosuppressive functions and metabolic reprogramming of MDSCs in glioblastoma and discuss their metabolic pathways as potential therapeutic targets to improve current incurable glioblastoma treatment.

## INTRODUCTION

### Classification of glioma and glioblastoma multiforme (GBM)

Gliomas are tumors of the central nervous system (CNS) that originate from transformed neural stem cells or progenitor glial cells [1]. The World Health Organization (WHO), based on histopathological characteristics and tumor's invasion patterns, divided gliomas into four groups: low-grade gliomas (grades I and II) are benign, well-differentiated, slow-growing tumors, whereas high-grade gliomas (grades III and IV) are poorly differentiated or anaplastic, rapidly proliferating, and strongly infiltrate brain parenchyma [1-4]. Among gliomas, grade IV GBM is the most frequent and aggressive astrocytic tumors and very difficult to treat due to the frequent mutations and dysfunctions of tumor suppressors or oncogenes such as *EGFR, PDGFRA, PTEN, TP53, NF1, CDKN2A/B*, and *TERT* promoter, and highly diffusive growth, which makes tumor resection challenging and contributes to rapid tumor recurrence [1].

Glioblastoma are currently classified into three distinct subtypes (proneural, classical, and mesenchymal), based on gene expression profile and prevalence of driver gene mutations [1, 3, 4]. Glioblastoma of the neural subtype are recently recognized as tumors with excessive adjacent neural tissue and this subtype, thus, is currently excluded from the class [4]. The pro-neural subclass of glioblastoma is further subdivided into two groups, those characterized by overexpression of *PDGFR*α and loss of the *p53* tumor suppressor gene and those with recurrent mutations within the genes coding for two isocitrate dehydrogenases (*IDH1* and *IDH2*). The latter is associated with a global hypermethylated genome (glioma CpG island methylator pheno-type (G-CIMP)). IDH mutant patients tend to have significantly prolonged survivals, when compared to non-G-CIMP IDH wild-type pro-neural glioblastomas [1, 4].

There are no specific treatment modalities based on a given subtype or mutation status of glioblastoma. All patients are given a standard of care treatment that consists of debulking surgery, followed by concomitant fractionated radiation and temozolomide (TMZ) chemotherapy followed by adjuvant TMZ [4]. The effect remains only palliative and the median survival time of adult patients with glioblastoma is only 15 months and <3% of patients survive longer than 5 years after diagnosis [1, 4]. Currently, multiple clinical trials including immunotherapy to improve the survival of glioblastoma patients are being tested, but the effect is not proven yet.

### Cellular components of the glioblastoma microenvironment

The tumor microenvironment (TME) is a dynamic structure in which various cells interact with tumor cells. In addition to cells, TME also contains various soluble factors, signaling molecules, extracellular matrix, and mechanical cues that can initiate neoplastic transformation, support tumor growth and invasion, protect the tumor from host immunity and foster chemotherapy resistance [5].

Compared to other solid organ tumors, gliomas have several unique differences in aspects of anatomy and physiology. First, in addition to tumor, glioma TME include stromal cells mainly represented by infiltrating peripheral immune cells, unique resident cells in the CNS such as microglial cells and reactive astrocytes, and endothelial cells, fibroblasts and pericytes [5-7]. Secondly, the blood–brain barrier (BBB) of the CNS uniquely serves to keep the CNS as an immune-privileged environment, where cells of the peripheral immune system are obstructed from entry in a normal situation. However, acute and chronic insult states such as infections, traumatic brain injury, neuroinflammation, or tumors in the CNS, can produce perturbation to the BBB. The breakdown of the BBB in glioma has been well-documented because brain tumors secret high concentrations of soluble factors such as vascular endothelial growth factors (VEGF) and matrix metalloproteinases (MMP), which compromise endothelial tight junctions, degrade proteoglycans in the surrounding extracellular matrix, and then allow the infiltration of various immune cells and blood-derived factors [3, 8, 9]. In addition, other studies have shown that the CNS is not tightly closed from increasing observations of systemic immune responses to CNS antigens, which likely travel via defined subarachnoid routes and to the cervical lymph nodes via the nasal mucosa [10, 11]. A recent interesting study showed that there is a direct vascular channel between the skull bone marrow and the brain surface and provided a new evidence for migration of peripheral myeloid cells [12]. Currently, these studies changed the concept of the CNS as an immune-privileged environment to a systemically restricted, but open system.

During glioblastoma progression, glioma cells secrete numerous chemokines and other factors that promote infiltration of CNS cells such as microglia, pericytes and endothelial cells, and blood-derived immune cells to the tumor. Here, the blood-derived immune cells include myeloid lineage cells such as monocytes/macrophages, leukocytes, MDSCs, DCs, and lymphocytes, mostly CD4^+^, CD8^+^ T, regulatory T cells (Treg), and NK cells [1]. Locally produced cytokines, chemokines, growth factors, and their crosstalk with components of the extracellular matrix re-educate infiltrating immune cells to acquire distinct functional properties, thus directing the immune system into either pro-inflammatory or anti-inflammatory responses in the glioblastoma. These immune cells also create a specific niche within the TME, which plays important roles in antitumor responses, immunosuppression, glioma growth, metastasis, and response to drug treatment [1, 4, 13, 14].

It has been shown in multiple studies using immuno-histochemistry and flow cytometry-based analyses that the majority of infiltrating immune cells in the glioma are microglia and blood-derived macrophages, collectively termed GAMM (glioma-associated microglia/macrophages), and MDSC in various intracranial xenograft mouse and genetically modified (GEM) mouse models and human glioblastoma patients [15-17]. In the transgenic Ntv-a/RCASPDGFβ tumor model in which RFP^–^/GFP^+^ microglia and RFP^+^/GFP^low^ macrophages/monocytes were isolated from tumors, flow cytometry studies demonstrated that microglia and macrophages/monocytes were recruited in the gliomas at the frequencies of 14% and 8.5%, respectively, and a similar proportion of GAMMs (16% microglia and 6.5% macrophages/monocytes) was observed in GL261 xenograft mouse tumors [13, 15, 16]. Comparably, another study in the same GEM mouse of glioma demonstrated that MDSCs constituted about 8% of the total tumor cell mass and mostly consisted of the CD11b^+^/Gr-1^low^ monocytic subset rather than the CD11b^+^/Gr-1^high^ granulocytic sub-set at the tumor site [17]. Clinical glioblastoma patient studies also showed the same extensive infiltration of these two types of myeloid cells into glioma [17-21]. These human studies demonstrated that intratumoral density of GAMMs and MDSCs increases according to glioma grade and correlates with prognosis of malignancy, implicating the crucial role of these myeloid lineage cells in immuno-suppression and glioblastoma progression [1, 17, 18, 22].

## IMMUNE RESPONSES IN THE GLIOBLASTOMA MICRO-ENVIRONMENT

### Anti-tumor responses

#### Tumor-infiltrating CD8^+^ cytotoxic T cells

The glioma microenvironment is infiltrated with small numbers of T lymphocytes, mostly CD4^+^ T helper (Th), CD8^+^ T cytotoxic (Tc), and CD4^+^CD25^high^FoxP3^+^ Treg cells [1]. Among these CD8^+^ Tc cells that express the transcription factors *Eomes* and *T-bet* are destined to develop into cytotoxic effector cells that produce IFNγ, granzyme B, and perforin and play the most important role in antigen-specific anti-tumor responses. A significant correlation between increased intratumoral numbers of CD3^+^ and CD8^+^ T cells and prolonged patient survival has been observed in different types of cancers [18]. Similarly, glioblastoma patients with intermediate or extensive CD8^+^ T-cell infiltrate at the time of diagnosis were more likely to have long-term survival than patients with rare or focal CD8^+^ T-cell infiltrates [1, 23]. A large neuropathological study also showed that infiltrating CD8^+^ T cells histologically in patients with newly diagnosed glioblastoma correlates with long-term clinical survival (> 403 days) [24]. For infiltration mechanism of CD8^+^ Tc cells, a study using immunohistochemical analysis of WHO grade IV glioblastoma provided a clue that infiltrating CD8^+^ Tc cells first bind to endothelial cells through cell adhesion molecules, and then infiltrate into the glioma [1, 24]. In this study, CD8^+^ Tc cells were frequently accumulated in glioblastoma fibrinogen positive areas, indicating the diffusion of fibrinogen due to leaky BBB vessels. This observation supports a mechanistic hypothesis that leaky vessels, which typically occur in glioblastomas, may facilitate T-cell transmigration [24].

#### NK cells

Natural killer (NK; characterized as CD3^−^CD56^+^CD16^+^) cells are highly effective cytotoxic lymphocytes in the innate immune response [4]. The activation of NK cells is tightly regulated by a sophisticated network of an activating receptor such as NKG2D, inhibitory receptors including killer cell immunoglobulin-like receptors (KIR), and immunoglobulin-like transcript/leukocyte immunoglobulin-like receptors (ILT/LIR) on NK cells [1, 4]. This network allows NK cells to distinguish normal from abnormal cells and target cell lysis through perforin-rich and granzyme-rich granules, when activating signals exceed inhibitory signals. Normal cells express major histocompatibility complex (MHC) I molecules, which interact with NK cell inhibitory receptor KIR and inhibits self-recognition and effective NK cell-mediated killing. In glioma, neoplastic cells also express MHC I and are therefore protected from recognition and destruction from NK cells [4].

Poli *et al*. reported that NK cells account for only a minor part of infiltrating CD45^+^ cell population in glioblastomas. Additionally, they showed that the infiltrating NK cells are somehow non-functional, possibly due to the contact with immunosuppressive cells, such as GAMM?s, MDSCs, and Tregs [25]. These immunosuppressive cells suppress cytotoxic activities of NK cells by suppressing the expression of NKG2D activating receptor and production of INFγ by TGFβ1 [26]. Kmiecik *et al*. identified NK cells (2.11 ± 0.54%) in eight glioblastoma biopsies. NK cells from these glioblastoma were identified as the CD56^dim^CD16^−^ pheno-type and only 57.45 ± 12.05% of them expressed NKG2D. The identity and function of the unusual CD56^dim^CD16^−^ NK subset has not been characterized, but it appears that they are functionally suppressed and phenotypically modified [27, 28]. These studies showed that immunosuppressive cells, although what cells actually suppress NK cells remains to be identified, control NK cell activity in glioblastoma. Hence, inhibiting immunosuppressive cells and enhancing NK cell activity would be a promising targeting strategy for further improvement of glioblastoma treatment.

### Suppression of anti-tumor responses in glioblastoma

Like many other non-CNS malignant cancers, gliomas develop multiple strategies by different immune cells to inhibit host antitumor responses. Accumulation of immuno-suppressive GAMMs, MDSCs, and Treg and their functional polarization into pro-invasive cells give more deleterious effects on anti-tumor responses in gliomas [13].

#### Glioma

Incomplete T-cell activation in the glioma microenvironment is also due to the fact that anti-tumor T-cell responses are also suppressed by cytokines TGF-β and IL-10 produced by glioma cells [29]. Thus, glioma cells can directly inhibit T cells by immunosuppressive cytokines and promote recruitment and expansion of immunosuppressive cells such as GAMMs, Treg, and MDSCs, which maintain inhibition of anti-tumor T and NK cells. In addition, glioma cells also lack B7.1/2 (CD80/CD86) co-stimulatory molecules, but overexpress B7-H1 (or PD-L1) co-inhibitory molecule for T cells. PD-L1 expressed in glioma cells was shown to strongly inhibit CD4^+^ and CD8^+^ T-cell activation through interaction with PD-1 on T cells. In the study, anti-B7-H1 neutralizing antibody was used for the blockade of PD-1/PDL-1 interaction, which increased cytokine production (IFNγ, IL-2, and IL-10) and the expression of CD69, the T-cell activation marker, by T cells [30].

#### GAMM

By far, the majority of immune cells within gliomas constitutes mostly two infiltrating macrophages, CNS resident microglia and blood-derived infiltrating macrophages, collectively called goes up to about 30-40% of the tumor mass [31, 32]. GAMMs have been known to play an important role in several immunosuppression mechanisms along with other immune cells including MDSCs and Treg. Under the influence of glioma-associated cytokines, GAMMs can upregulate immunosuppressive PD-L1 [33, 34], which promotes T-lymphocyte anergy as well as Fas ligand (FASL), which promotes T-lymphocyte apoptosis [35]. Moreover, gliomas cause GAMMs to substantially decrease the expression of MHC molecules and pro-inflammatory cytokines including TNF-α, while increasing the activation of the transcription factor STAT3 likely through S100B-receptor for advanced glycation end products (RAGE) axis [29, 36]. STAT3 activation by GAMMs promotes the secretion of immunosuppressive cytokines, IL-6 and IL-10, which are known to inhibit cytotoxic T lymphocyte function, among other immunosuppressive actions [29, 37, 38].

In addition, GAMMs can closely cross-talk with glioma for glioma invasion in a sequential manner. First, infiltrating tumor-associated macrophages (TAM) release several factors such as TGFβ, stress-inducible protein 1 (STI1), epidermal growth factor (EGF), IL-6 and IL-1β to promote glioma cell invasion. Secondly, microglia also release TGF-β, which triggers the release of pro-MMP2 from glioma cells. Pro-MMP2 is then cleaved into active MMP2 by microglia-expressed MT1-MMP. Microglial MT1-MMP expression is stimulated by versican, which is released from glioma cells. Versican activates TLR2 and p38-MAP-kinase signaling in microglial cells, which leads to MT1-MMP up-regulation and triggers MMP9 release. MMP2 and MMP9 at the final step break and open the extracellular matrix to promote tumor invasion [31]. These sequential processes support the building of extracellular matrix in the boundary of glioma and thus, assist glioma invasion [31].

Interestingly, Ye *et al*. showed that GAMMs can enhance the invasion of glioma stem cells (GSC) via TGFβ1 signaling mechanism [39]. Zhou *et al*. showed that periostin secreted by GSC recruits M2-type glioma-associated macrophages (GAM) and promotes malignant growth [40]. In addition, Guo *et al*. showed that hypoxia promotes glioma-associated macrophage infiltration via periostin from GSCs and subsequent M2-type GAM polarization by upregulating TGF-β and M-CSFR [41].

#### MDSC

Although the functions of MDSCs are well described in different types of cancers, information regarding detailed mechanism of immunosuppressive functions of MDSCs in glioblastoma compared to GAMMs has not often been addressed and their characterization remains mainly descriptive [1, 4, 29]. In addition, studies on cellular interaction and immune regulation between MDSCs and gliomas and immunosuppression of anti-tumor lymphocytes including T and NK cells systemically and at glioblastoma sites are currently limited [13, 17, 20]. Despite of lack of studies on immunosuppressive functions of MDSCs, there have been several important mechanistic evidences of immunosuppression functions to target anti-tumor T and NK cells by MDSCs in glioblastoma.

First, accumulation of MDSCs in peripheral blood of glioblastoma patients induces immunosuppression of T cells as well as NK cells. Increased plasma levels of arginase 1 and G-CSF secreted from MDSCs contribute to enhancement of MDSC suppressor function and its accumulation in glioma sites [20]. In the same study, T cells isolated from patients with glioblastoma had significantly depressed IFNγ production following stimulation [20]. Subsequent depletion of polymorphonuclear (PMN) -MDSCs from peripheral blood using anti-CD33/CD15-coated beads monocytic- (M-) MDSCs of glioblastoma patients increase levels of intracellular and serum S100A8/9 levels compared with M-MDSCs in healthy controls, which correlates with increased Arg1 activity in serum [13, 42].

Secondly, in a study with 52 glioblastoma patients, it was shown that there is a correlation between the number of PMN-MDSCs and CD4^+^ effector memory T-cells (CD4^+^ Tem) within the gliomas. Tumor-derived CD4^+^ Tem expressed high levels of PD-1, indicating that they are functionally exhausted. The expression of PD-L1 was also significantly up-regulated on glioma-associated MDSCs. Their findings provide an evidence for the accumulation of different MDSC subsets in glioblastoma patients and suggest that PMN-MDSCs in peripheral blood and at the tumor site may participate in glioblastoma-induced T-cell suppression by PD-1/PD-L1 checkpoint inhibition mechanism [1, 43].

Thirdly, Otvos *et al*. showed recently that macrophage migration inhibitory factor (MIF), which was produced at high levels by GSC, increased the expression of the arginase-1 in MDSCs in a chemokine receptor CXCR2-dependent manner. Reduction of MIF conferred a survival advantage to tumor-bearing animals and increased the cytotoxic T cell response towards the tumor [44]. This report showed a particular example of hierarchical regulation that GSCs first modulate the functions of MDSCs, which subsequently regulate anti-tumor CD8^+^ Tc cells within the TME.

#### Treg

Treg (CD3^+^CD4^+^CD25^high^FoxP3^+^) cells are known as potent suppressors of the deleterious adaptive immune responses by inhibiting the proliferation of effector CD4^+^ Th cells [1]. Accumulation of Treg in different types of cancers has been implicated with poor prognosis and higher grade of malignancy [45]. Although studies have shown increased infiltration of Treg cells to tumor of glioblastoma patients when compared to healthy controls and an increase in signature gene expression of Treg markers such as *Foxp3, CD25, CTLA-4*, and *GITR* by gene-profiling analysis, different laboratories have shown unmatched results on the frequencies of Treg cells by flow cytometry and immunohisto-chemistry [24, 28, 46]. Thus, information on immunosuppressive functions by Treg cells in glioblastoma and the prognostic implication of Treg accumulation in patients with glioblastoma remains currently to be determined. Further standardized quantification of Treg frequencies and clearer dissection of heterogeneous intratumoral T cells in glioblastomas may be of critical importance for clinical prognosis and the design of future immunotherapies [1].

## GENERATION AND PHENOTYPIC DEFINITION OF MDSCs

Numerous publications have reported a strong correlation between the development of chronic inflammatory conditions such as tumor, infections, autoimmune disorders, and shocks and expansion of MDSCs [47-50]. MDSCs are initially generated in the bone marrow (BM) from common myeloid progenitor cells. They are known as immature myeloid populations that fail to differentiate terminally into mature myeloid cells [51, 52]. Multiple cancer-associated factors secreted from tumor and tumor stromal cells can mediate the generation and expansion of MDSCs and they inhibit the development of terminally differentiated myeloid cells such as granulocytes, macrophages, and DCs [48, 49, 53].

### Phenotypic definition and functional differences of MDSC subsets

In humans and mice, MDSCs can be mainly divided into two subsets, granulocytic (G) MDSCs (G-MDSCs; also named PMN-MDSCs) and monocytic (M) MDSCs (MMDSCs), on the basis of cell morphology. In addition to the morphology, various phenotypic markers that distinguish the two subsets have been identified [54-56]. In humans, PMN-MDSCs are defined as CD11b^+^CD33^+^CD14^-^CD15^+^HLADR^-/low^CD66b^+^, whereas M-MDSCs are defined as CD11b^+^CD33^+^CD14^+^CD15^-^HLA-DR^-/low^. In mice, PMN-MDSCs are phenotypically CD11b + Ly6C lo Ly6G^+^^Gr1^bright^CD49^-, while M-MDSCs are CD11b + Ly6C hi Ly6G^-^^Gr1^low^F4/80^+ ^CD49^+. In human, early-stage MDSC (e-MDSC) are described as Lin^-^(CD3^-^CD14^-^CD15^-^CD19^-^CD56^-^) HLADR^-^CD33^+^ because this Lin^-^ population contains more immature MDSC progenitors, but mouse counterpart of e-MDSC is not defined yet [56].

These two MDSC subsets suppress immune responses through different mechanisms: PMN-MDSCs suppress antigen-specific CD8^+^ T cells mainly by producing reactive oxygen species (ROS), whereas M-MDSCs express inducible nitric oxide synthase (iNOS or NOS2) and arginase 1 (ARG1) that deplete L-arginine in the local environment and block translation of the T cell CD3 zeta chain, and generate reactive nitrogen species (RNS) that inhibit T cell receptor (TCR) signaling and promote T-cell apoptosis in no antigen-specific manner [1, 49].

## EXPANSION AND MIGRATION OF MDSCs IN GLIOBLASTOMA

### Location dependent suppression of MDSCs in glioblastoma

The numbers of MDSCs in various cancers including glioma are frequently increased in blood, spleen, and tumor mass, and they correlate with cancer stage, metastasis, and chemotherapy response [1]. In human glioblastoma studies, intratumoral and systemic blood MDSC density also increases together during glioma progression and correlates with the grade of glioma malignancy [1]. Raychaudhuri *et al*. reported that patients with glioblastoma have elevated levels of MDSCs in blood when compared with age-matched healthy donors, suggesting an increase of MDSC frequency in blood as a potential clinical biomarker. The majority of the MDSCs in glioblastoma patients were CD15^+^CD14^−^ PMN-MDSCs (82%), followed by lineage-negative e-MDSCs (15%) and M-MDSCs (3%) [20]. Gielen *et al*. also reported increased percentages of both PMN-MDSCs and M-MDSCs in the blood of glioblastoma patients when compared with healthy donors. They showed that during progression from the low-grade (II-III) to the high-grade IV glioblastoma, their frequencies in blood gradually increase and correlate with poor prognosis. MDSCs consisted almost exclusively of CD15^+^ PMN-MDSC cells. Immunohistochemistry also confirmed infiltration of glioma tissues with CD15^+^/HLAII^–^ cells [1, 21]. Another study with 52 glioblastoma patients also revealed a significantly higher frequency of CD15^+^ CD14^low^ PMN-MDSCs and CD15^+^ CD14^high^ M-MDSCs in blood and tumor sites, when compared with healthy controls [43]. These studies show expansion of both PMN- and M-MDSC subsets in glioblastoma patients. Although a PMN-MDSC subset is the dominant subset in peripheral blood and at the tumor site, it appears that both PMN- and M-MDSC subsets participate in a cooperative manner in T-cell suppression [1, 42, 43]. In addition to human studies, the expansion of murine MDSCs was also confirmed in GEM mouse models and a rat C6 glioma model, but the mechanism how MDSC suppresses T cells remains to be determined [17, 57].

The mechanism of immunosuppression by MDSCs is regulated in a location dependent manner in various cancer models. In peripheral lymphoid organs, MDSC-mediated suppression of CD8^+^ T cells requires the presentation of antigens by MHC class II of MDSCs and direct antigen-specific MDSC–T cell contact. By contrast, at the tumor site MDSCs are able to suppress nearby T cells in both antigen-specific and non-antigen-specific paracrine manner [47, 58, 59]. In addition, MDSCs in blood also can suppress T cells in non-antigen-specific paracrine manner through up-regulation of ARG1 induced by serum S100A8/A9 [42]. This multiple suppression activities in different locations (both systemically and at tumor sites) by MDSCs can exacerbate anti-tumor response more extensively in tumors [48]. The mechanisms under suppression of T cell functions by MDSCs in blood and within the glioblastoma are currently not fully understood [1, 4], but, as described above, expansion of MDSCs in multiple locations including peripheral lymphoid tissues, blood and glioma sites suggest that they should synergistically and efficiently suppress anti-tumor T cell response in human glioblastoma patients.

### Recruitment of MDSCs into the tumor site and glioblastoma

During chronic inflammation such as tumors, MDSCs are expanded in multiple locations such as BM, blood, and peripheral lymphoid tissues such as spleen, and then recruited to local tumor sites and are circulated between tumors and peripheral lymphoid tissues [60, 61]. One of important factors to direct the migration of MDSCs to glioma sites is the chemokines. The unique distribution of chemokines in the TME determines the recruitment of different MDSC subset (M or PMN) and seems to be dependent on the tumor models [53, 62].

#### CCL2/CCR2

Studies have shown that unique combination of multiple chemokines including CXCL1, CXCL8, CXCL12, CCL1, CCL3, CCL5, CCL7, and CX3CL1 differently regulate the recruitment of MDSCs in different cancer models [63-65]. Among multiple chemokines the role of chemokine (C-C motif) ligand (CCL)2 and its receptors in the attraction of M-MDSCs in different cancer models has been extensively described [66]. In particular, it has been demonstrated that an accumulation of M-MDSCs in several mouse tumor models including glioma occurred via an interaction between CCL2 and its receptors, chemokine (C-Cmotif) receptor (CCR)2, 4, and 5 [53, 67].

In a GEM mouse model of glioma, it has been recently demonstrated that CCL2-CCR2 interaction plays a critical role in the recruitment of M-MDSCs to the tumor site, in which CCL2 is produced by GAMMs within the glioma microenvironment [62, 68]. In this murine model, CCL2 production by GAMMs was induced by tumor-derived CCL20 and osteoprotegerin. The same study also showed that in a mixed BM chimera experiment CCR2-deficient M-MDSCs were defective in glioma accumulation. Collectively, this study proved the critical role of CCR2 in migration to glioma with a result that gliomas in CCL2-deficient mice displayed reduction of both Treg and monocytic MDSCs infiltration [62].

## IMMUNO-SUPPRESSIVE FUNCTIONS OF MDSCs

**Figure 1:**
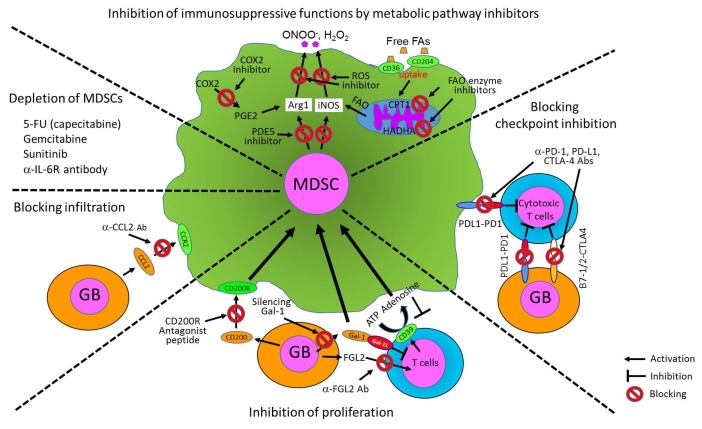
FIGURE 1: Immunosuppressive functions of MDSCs in the tumor microenvironment. The cartoon shows cellular interactions of MDSCs with tumors and immune cells and key cellular factors produced by MDSCs and tumors that promote an immunosuppressive environment both systemically and within the tumor. MDSCs can suppress anti-tumor functions of immune cells, but promote other immunosuppressive cells such as Treg and TAM as well as tumor growth and metastasis. The key cellular factors include cytokines, chemokines, surface receptors, enzymes, and metabolites.

MDSCs have been known as major suppressor cells for T cells through multiple mechanisms, particularly inhibiting anti-tumor activity of cytotoxic T cells. They also suppress other immune-activating cells including DCs, pro-inflammatory macrophages, and NK cells, but promote activation of immunosuppressive Treg. Moreover, MDSCs can directly promote tumor angiogenesis, invasion, and metastasis [47, 49, 53, 55]. The fact that MDSCs can suppress multiple types of immune-activating cells has drawn a lot of interests to the field of cancer and other chronic inflammatory diseases and emphasized the importance of MDSCs as a therapeutic cellular target in various disease models. Here, we summarize the immunosuppressive functions of MDSCs to different immune cells in detail in the following section. We also illustrate cellular interactions of MDSCs with tumors and immune cells and key cellular factors produced by MDSCs and tumors that promote immunosuppressive environment both systemically and within the tumors (**Fig. 1**).

### T cell suppression

MDSCs can directly suppress T cell functions through multiple mechanisms. First, MDSCs induce oxidative stress by releasing either ROS or RNS. Peroxynitrite (as an RNS product) and hydrogen peroxide are produced by the combined and cooperative activities of NADPH oxidase (NOX), ARG1 and iNOS (NOS2) in different MDSC subsets. These reactive species can drive several molecular blocks in T cells, ranging from the loss of TCR ζ-chain expression and interference with IL-2 receptor signaling to the nitration and subsequent desensitization of the TCR, thus promoting apoptosis of T cells [47]. Secondly, MDSCs deplete L-arginine by iNOS, Arg1, and arginine transporter (Cat2) and L-cysteine via its consumption and sequestration mechanism in the TME, which inhibits T cell growth and induce apoptosis [47, 69]. Thirdly, HIF1α produced by hypoxia in the TME induces PD-L1 on MDSCs as well as TAMs, which inhibit activation of T cells through co-inhibitory receptor interactions (immune checkpoint mechanism) [70]. Fourthly, MDSCs can inhibit the migration of CD4^+^ and CD8^+^ T cells. In this mechanism, CCL2 is nitrated or nitrosylated by RNS such as peroxynitrite in the tumor environment. While immature myeloid cells are attracted by modified CCL2, effector CD8^+^ T cells are not recruited by modified CCL2, which may explain the selective enrichment of myelomonocytic cells within mouse and human tumors [71].

### Treg activation and differentiation

MDSCs can also promote the clonal expansion of antigen-specific Treg cells and induce the conversion of naive CD4^+^ T cells into induced Treg cells [1]. The mechanisms are not fully understood, but may involve cell-to-cell contact through CD40–CD40L interactions, the production of soluble factors such as IFNγ, IL-10 and TGFβ, and possibly the expression of ARG1 by MDSCs [47]. Interestingly, human CD14^+^HLA-DR^low/^– MDSCs was shown to promote the trans-differentiation of Th17 cells into FOXP3^+^ induced Treg cells by producing TGFβ and retinoic acid [47, 72].

### NK cell regulation

MDSCs can decrease the number and suppress the function of NK cells [47]. Li *et al*. showed that membrane-bound TGFβ1 by MDSCs induces consequent down-regulation of activating NK receptor NKG2D expression and decreased IFNγ production, thus lowering cytotoxic activity [73]. Hoechst *et al*. showed that MDSCs suppress autologous NK cell cytotoxicity and cytokine secretion through interaction with the NK cell receptor NKp30 (also known as NCR3) [74]. Both studies showed that the suppression needs cell contact between MDSCs and NK cells.

### Macrophage and DC regulation

MDSCs can modulate functions of macrophages and DCs. MDSC potency is increased by inflammation, which enhances the crosstalk between MDSCs and macrophages. Through an IL-10- and cell contact-dependent mechanism, MDSCs skew macrophages towards an M2-type macrophage phenotype by decreasing macrophage production of IL-12, leading to an immunosuppressive environment [47, 75]. On the contrary, MDSCs impair DC function by producing IL-10, which inhibits maturation of fully activated DCs and TLR-induced IL-12 production by DCs, and thus ultimately suppress DC-mediated activation of T cells in hepatocellular carcinoma [47, 76].

### Pro-tumor ability: angiogenesis and metastasis

MDSCs can promote growth of tumors (or cancer stem cells) by directly supporting angiogenesis and metastasis through angiogenic factors such as VEGF and basic fibroblast growth factor (bFGF), MMPs, and cytokines [53, 55]. Several studies showed that MDSCs promote tumor invasion and metastasis by two mechanisms: 1) elevated production of multiple MMPs, which play a major role in matrix degradation, release of VEGF-A, and chemokines to create a pre-metastatic environment, and 2) fusion of MDSCs with tumor cells to promote the metastatic process [53, 77, 78].

## METABOLIC REPROGRAMMING OF MDSCs IN THE TME

**Figure 2:**
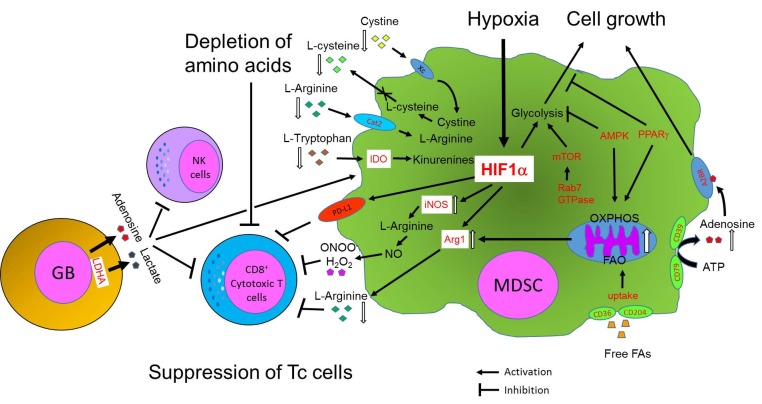
FIGURE 2: Immune suppressive functions of MDSCs are regulated by metabolic pathways in the tumor and glioblastoma (GB) microenvironment. The cartoon summarizes three major metabolic pathways governed by MDSCs: 1) FAO pathway through PPARγ and AMPK, 2) glycolysis through HIF1α and mTOR, 3) depletion of amino acids by enzymes and transporters. These metabolic pathways determine the fate and the immune functions of MDSCs. MDSCs and tumors also cooperatively control survival and anti-tumor functions of NK and T cells by tightly regulating levels of metabolites such as adenosine, lactate, toxic ROS or RNS, and amino acids in the TME. Factors in red indicate key regulators of metabolic pathways for immunosuppressive functions of MDSCs.

Although there have been numerous studies describing MDSC metabolism in various cancer models, information on the metabolic reprogramming of MDSCs in glioblastoma is currently scarce [1]. Here, we review the metabolic reprogramming of immune cells, focusing on MDSCs, with different types of cancer models and a few cases of glioblastoma studies. In this section, we mainly focus on the metabolic reprogramming of tumor cells and MDSCs and immuno-regulation of MDSCs by metabolites produced in tumor environments. Here, we also illustrate major metabolic pathways preferentially selected by MDSCs and their key intracellular and extracellular metabolites from current literature, showing how metabolic pathways and metabolites from tumors and glioblastomas determine the fate and immunosuppressive functions of MDSCs (**Fig. 2**).

### Metabolic reprogramming of cells in the TME

Metabolism has not been appreciated until recent years as an important driver of tumorigenesis and immune responses in the TME [79, 80]. Tumor cells must adapt to their bioenergetic and biosynthetic demands to support rapid proliferation, survival, and differentiation. To do so, they prefer to use glycolysis for rapid growth, as opposed to normal cells, which mainly rely on mitochondrial oxidative phosphorylation (OXPHOS). Even under aerobic conditions, they choose a glycolytic pathway (aerobic glycolysis), which is known as the Warburg effect [81]. The Warburg effect is thus a useful adaptation of tumor cells for maintaining their growth and survival and is a hallmark of cancer progression.

By reprogramming their metabolism, tumor cells can adapt to metabolic needs in the TME. In addition, recent accumulating studies have shown that infiltrating immune cells (e.g., TAMs, tumor-associated DCs, MDSCs, neutrophils, T cells, B cells, and NK cells) also undergo metabolic reprogramming to compete with tumor and other cells for nutrients and to survive in a harsh tumor environment such as oxygen and nutrients deprivations [83, 84]. Furthermore, several recent studies showed that different immune subpopulations of myeloid cells and lymphocytes also reprogram their metabolism to meet their metabolic needs [52, 80, 85].

Overall, these studies drew two major conclusions: 1) a metabolic dialog between tumor cells and the immune cells, and 2) a close link between metabolic reprogramming of immune cells and their plasticity in immune functions during tumor growth [83]. This metabolic reprogramming of cells in the TME has recently been an active focus in the tumor immunology field [52, 82, 83, 85-87].

### Regulation of MDSC functions by metabolic reprogramming in cancer

A current understanding of immune suppression mechanisms in tumor is that immune cell functions are biased and misdirected through metabolic reprogramming caused by the TME. Numerous evidences have suggested that tumor cells actually manipulate immune cells by reprogramming the metabolic status of protective immune T and NK cells to anergy or cell death, converting the functions of myeloid cells to immunosuppressive phenotypes, or further supporting tumor growth [83].

Multiple papers suggest that similar to other immune cell types the phenotypic heterogeneity of tumor-associated MDSCs is also under the control of metabolic and inflammatory parameters such as oxygen, nutrient, metabolite levels, and polarized inflammatory programs [87]. Particularly, nutrients in the TME are essential for survival and functions of infiltrating immune cells. Lack of or decreased levels of nutrients limit their survival and induce apoptosis of immune cells in the TME. One good example is the suppression of T cells through the depletion of amino acids by MDSCs. MDSCs can deplete amino acids by several mechanisms regulating the levels of amino acids and thus, determine the fate, growth, and immune functions of cytotoxic CD8^+^ T cells in the TME (**Fig. 2**).

Multiple studies have shown that tryptophan, L-arginine, and cysteine are depleted by metabolic enzymes, Arg1, iNOS, and indoleamine 2,3 dioxygenase (IDO), and amino acid transporters such as cysteine-glutamine anti-porter (Xc) and cationic amino acid transporter 2 (Cat2) of MDSCs, thus limiting growth and immune functions of T cells [88-90]. It is particularly interesting that IDO from MDSCs produces the immune-regulatory metabolite kynurenine, which can interact with the aryl hydrocarbon receptor expressed by T cells, DCs, and Treg, thereby regulating immune functions [91]. Kynurenine in the TME was also shown to favor conversion of naïve CD4^+^ T cells into Treg [92].

On the other hand, the inflammatory programs of MDSCs sense these environmental parameters and allow MDSCs to respond and select the most efficient metabolic pathways such as glycolysis, OXPHOS, salvaging TCA (tricarboxylic acid) cycle, and lipid metabolism according to their metabolic needs. For the past years, several research groups have demonstrated that the development and the immune functions of MDSCs are regulated particularly by lipid metabolism. MDSCs sense lipid metabolites produced by the TME, which particularly enhance immunosuppressive functions of MDSCs [87, 93, 94]. In addition, studies by Ding *et al*. showed another metabolic aspect of MDSCs that the decision to choose between lipid and glucose metabolism by MDSCs is tightly controlled by two signaling complexes such as PPARγ and mTOR, which sense extracellular glucose and metabolite status. Such decision determines the development and immunosuppressive functions of MDSCs [95-97].

#### Fatty Acid Oxidation (FAO) and lipid metabolism

Recent several studies by Ochoa's laboratory have shown evidence that tumor-associated MDSCs uniquely opt FAO and subsequently, enhance immunosuppression [87, 93, 94]. Hossain *et. al*. showed that accumulated fatty acids in the TME enhance immunosuppressive functions of tumor-associated MDSCs [93]. The mechanism under this is that MDSCs uptake fatty acids mediated by CD36 and CD204 and initiate the FAO pathway. Tumor-associated MDSCs, both granulocytic and monocytic subsets, preferentially choose FAO over glycolysis as a primary source of energy and undergo metabolic reprogramming that enhance mitochondria protein function such as electron transfer complex system and TCA cycle. This coincides with increase of mitochondrial biogenesis (mass), key FAO-associated genes (*CPT1, ACAD, PGC-1a, HADHA*), and oxygen consumption rate. As a result, switching to the FAO pathway enhances secretion of arginase 1 and NO and peroxynitrite (ONOO-) by MDSCs, thus resulting in inhibition of CD8 T cells.

Furthermore, they showed that FAO inhibition by several drugs restrains the metabolic reprogramming and immune-regulatory functions in tumor-infiltrating MDSCs. Actually, FAO inhibition by drugs resulted in a T cell-dependent delay in tumor growth and prevents immunosuppressive functions of MDSCs. Likewise, they showed that MDSCs in human patients also take up high amounts of fatty acids (FA) and increase the expression of FAO enzymes (*CPT1* and *HADHA*). In addition, a recent study showed that G-MDSCs overexpress lectin-type oxidized *LDLR-1*, which identifies a subpopulation of ER-stressed, immunosuppressive G-MDSCs in cancer patients, providing another evidence for FAO activation of MDSCs by lipid uptake in human [87]. Collectively, these studies suggested that tumor-associated MDSCs reprogram their metabolic pathway to adapt to unique tumor environments such as limited O_2_ and glucose, but high level of FA and thus, uniquely prefer to use lipids or FA as another energy source. However, any study on metabolic reprogramming to utilize fatty acids by MDSCs is currently not done yet in glioblastoma models.

#### LAL (lysosomal acid lipase) and mTOR

LAL is essential for the hydrolysis of cholesteryl esters and triglycerides to generate cholesterol and free FAs in cellular lysosomes. In humans, functional loss of the *lal* gene leads to two lipid storage diseases: Wolman disease and cholesteryl ester storage disease [98]. Ding *et al*. first observed that ablation of the *lal* gene (*lal*^-/-^) systemically increases expansion of CD11b^+^Ly6G^+^ MDSCs that caused myelopro-liferative neoplasms in *lal*^-/-^ mice [95]. In the subsequent studies they showed that in LAL-deficient mice the metabolic reprogramming of MDSCs is switched from the FAO pathway to glucose-dependent oxidative pathway, similar to the Warburg pathway [96]. This study demonstrated that the functional deficiency in FA release and FAO pathway causes MDSCs to switch metabolic reprogram to glycolysis, which enhances development of proliferative MDSCs, but inhibits immunosuppressive functions, suggesting that a metabolic pathway determines a decision between development and immune functions of MDSCs. This change was proven by gene microarray analysis showing that glucose transporters were significantly upregulated. In the study, deficiency of LAL somehow promoted downstream Rab7 GTPase protein, which was shown to physically interact with mTOR, a master regulator of glycolysis. In conclusion, they showed that LAL-deficiency (or FAO blockage) forces a metabolic switch towards mTOR signaling (glycolysis preference) of MDSCs and Rab7 GTPase is a critical upstream player for mTOR signaling in MDSCs' homeostasis and tumorigenic functions [95-97]. Similar to the mTOR pathway, they found that LAL function in neutral lipid metabolic signaling is mediated by PPARγ. Collectively, these studies demonstrated that mTOR and PPARγ in MDSCs are major regulators that sense environmental parameters such as glucose and lipid levels, respectively, and can switch between glycolysis and FAO pathways, depending on metabolic demands of the TME [97]. However, efforts on metabolic reprogramming to target mTOR or PPARγ by MDSCs are currently not done in glioblastoma models.

### Regulation of MDSC function by metabolites released from cancer cells

Here we discuss the regulation of MDSC function by metabolites released from cancer cells. Cancer metabolites can directly signal immune cells and regulate their metabolic and functional responses. They can be transported into neighboring immune cells and directly converged into metabolic pathways as precursors of anabolism or catabolism. We focus on two metabolites, lactate and adenosine, that are known to regulate the functions of MDSCs and provide insight into targeting MDSCs in cancers and glioblastoma.

#### Lactate

Lactate is massively produced by aerobic glycolysis in various cancers including glioblastoma [83, 99]. It is produced by lactate dehydrogenase A (LDHA) through HIF1α activation in a hypoxic environment. It has been recently shown that the lactate produced by tumor cells impairs immune effector cells by directly inhibiting the cytolytic functions of T cells or by polarizing immune responses toward a Th17/Th23 pro-inflammatory profile [52, 100, 101]. In addition, lactate is also known to stimulate generation of MDSCs and inhibit the maturation of DCs. Moreover, low pH by high lactate production strongly inhibits NK cells through histone deacetylase (HDAC) inhibition [52, 90]. However, it is unclear how MDSCs' expansion is regulated by lactate.

#### Extracellular adenosine

While intracellular adenosine is mainly used in energy metabolism, nucleic acid metabolism and extracellular adenosine also plays an important role in intercellular signaling [102]. Extracellular adenosine has been known as a modulator of immune functions. It can be an activator or an inhibitor, depending on the immune cell types. Its signal is transmitted by G protein-coupled adenosine receptors, which are composed of four known types (A1R, A2AR, A2BR, and A3R) [103]. It affects diverse physiological functions, including neurological, cardiovascular, and immunological systems [102, 103].

In addition, adenosine receptor signaling induces recruitment and regulates functions of immune-regulatory immune cells such as Treg, macrophages, and MDSCs [104]. FoxP3, a key transcriptional factor for the immunosuppressive activity of Treg cells, is inducible by A2A adenosine receptor (A2AR) stimulation. Similarly, A2B adenosine receptor (A2BR) plays a predominant role in the adenosine-dependent differentiation of macrophages into M2-type macrophages and expansion of MDSCs [102]. MDSCs uniquely express high levels of ATP hydrolases: CD39 (ectonucleoside triphosphate diphosphohydrolase 1, E-NTPDase1) / CD73 (ecto-5'-nucleotidase, Ecto5'NTase) [104]. MDSCs can produce extracellular adenosine within tumor lesions by these enzymes as an additional mechanism and thus exacerbate immune-suppression in an auto-crine manner.

In addition, adenosine can also influence the accumulation of MDSCs within tumors lesions [104]. Indeed, treatment of tumor-bearing mice with A2BR agonist increased numbers of MDSCs in tumors and accelerated tumor growth [90, 102]. In another study, A2BR deficiency in Lewis lung carcinoma-bearing mice induced both a low number of tumor-infiltrating MDSCs and reduced levels of intratumoral VEGF [105]. Correspondingly, it was shown that the blockade of A2BR with a selective antagonist reduces significantly the number of tumor-infiltrating MDSCs, inhibited tumor angiogenesis and thereby improves T cell-mediated immune surveillance in a melanoma model [104, 106]. Collectively, these results concluded that mechanistic cooperation between adenosine-generating enzymes and adenosine receptors in MDSCs within the tumor tissue can maintain their numbers and maintain immunosuppressive functions.

## THERAPEUTIC APPROACHES FOR CONTROL OF MDSCs

As MDSCs are one of the central immunosuppressive factors in cancer and other pathological conditions, various therapeutic strategies and new approaches that control the number and/or function of MDSCs *in vivo* have been explored [22, 49]. These approaches will undoubtedly uncover the biology of these cells and advance clinical treatment and new drug development for cancer, including glioblastoma as well as other pathological conditions. Here, we summarize current targeting strategies to control MDSCs.

### Promoting myeloid-cell differentiation

One of the most promising and simplest approaches to target MDSCs for therapy is to promote their differentiation into mature myeloid cells that do not have suppressive functions [49]. Vitamin A and its derivatives, retinoic acid and All-*trans* retinoic acid (ATRA), have been identified as compounds that can mediate this effect [49, 107]. Particularly, ATRA has been shown to induce the differentiation of MDSCs into DCs and macrophages *in vitro* and *in vivo*. Administration of therapeutic concentrations of ATRA showed a substantial decrease in the numbers of MDSCs in patients with cancer and tumor-bearing mice. The mechanism of ATRA-mediated differentiation involves dramatic upregulation of glutathione synthase in MDSCs accompanied by increased production of glutathione, which neutralizes ROS in MDSCs and drives the switch towards myeloid-cell differentiation [49, 108]. Some evidence also suggestes that vitamin D3 may be another compound that can decrease MDSC numbers by promoting myeloid-cell differentiation in patients with cancer [49, 109].

### Inhibition of MDSC expansion and tumor metastasis

Because MDSC expansion is known to be regulated by tumor-derived factors and MDSCs regulate angiogenesis and tumor metastasis, several studies have focused on neutralizing the effects of these factors between tumor and MDSCs. These include stem cell factor (SCF), VEGF, and MMP9 which have been experimentally proven [49, 110-113]. SCF is secreted from various carcinomas and promote the expansion of MDSCs. Inhibition of SCF-mediated signaling by either knock down of SCF or blocking with an anti-body against its receptor, KIT, can decrease both MDSC expansion and tumor angiogenesis [110]. VEGF is another tumor-derived factor and promotes MDSC expansion [49]. While VEGF-trap (a fusion protein that binds all forms of VEGF and placental growth factor) shows no change in the number of MDSCs in patients with refractive solid tumors, treatment of patients with metastatic renal-cell cancer with a VEGF-specific blocking antibody known as avastin showed a decrease in the frequencies of the CD11b^+^VEGFR1^+^ MDSCs in the peripheral blood [49, 112]. MMP9 is produced from carcinomas and MDSCs. Inhibition of the expression of MMP9 with amino-biphosphonate in tumor-bearing mice decreased the number of MDSCs in the spleen and tumor tissues and resulted in a significant delay in the growth of spontaneous NeuT tumors in transgenic BALB/c mice [49, 113]. However, the detailed mechanism of this outcome was not clearly elucidated. These studies showed close interaction between tumors and MDSCs and regulation by soluble factors derived from tumors, which can increase the expansion of MDSCs and promote tumor metastasis. Blocking the interaction of these stimulating factors would also be a potential approach for targeting MDSCs.

### Inhibition of MDSC migration to tumor

In syngeneic and intracranial xenograft mouse models with GL261 glioma, administration of an anti-CCL2 antibody could block recruitment and decrease the number of both MDSCs and GAMMs in the TME, leading to prolonged survival of tumor-bearing mice [22, 114].

### Elimination of MDSCs

MDSCs can be directly depleted in pathological settings by using chemotherapeutic drugs. Current anti-MDSC therapy is mainly to administrate minimum dose of cyclophosphamide, 5-fluorouracil (5-FU), or gemcitabine [49, 93]. Administration of gemcitabine to tumor-bearing mice resulted in a significant reduction in the number of MDSCs in the spleen and a marked enhancement in the anti-tumor response that was induced by immunotherapy. This effect was specific only to MDSCs, as there was no significant decrease in the number of B and T cells in these animals [115]. Interestingly, a recent study by Otvos *et al*. showed that 5-FU treatment significantly and selectively depleted MDSCs and successfully improved glioblastoma treatment and this therapy is being tested in a clinical trial (NCT02669173) [44]. In addition, several studies with the tyrosine kinase inhibitor sunitinib also showed reduction of MDSC numbers and their suppressive functions [93, 116]. Sumida *et. al*. showed that the anti-IL-6 receptor mAb can specifically eliminate MDSCs and inhibit tumor growth by enhancing T cell responses, thus showing a potential as an immunotherapeutic application [117].

### Inhibition of MDSC function through metabolic pathways

Another strategy to target MDSCs is to block the signaling and metabolic pathways that regulate the production of suppressive factors by these cells [49].

One such promising target is cyclooxygenase (COX) 2. It was shown that COX2 is required for the production of prostaglandin E2 (PGE2), which subsequently induces the up-regulation of *Arg1* expression by MDSCs in mammary carcinoma [49]. Accordingly, COX2 inhibitors such as acetylsalicylic acid or celecoxib were found to prevent production of PGE2, down-regulate the expression of *Arg1*, and delay glioma progression. They also reduced CCL2-mediated accumulation of CD11b^+^Ly6G^+^ G-MDSCs in both BM and the TME and increased the numbers of CD8^+^ T cells in a CXCL10-dependent manner in a colon cancer model. These results showed that COX2 inhibitors could improve the whole anti-tumor T-cell responses and enhance the therapeutic efficacy of immunotherapy [22, 49, 68, 118].

Similarly, phosphodiesterase 5 inhibitors, such as sildenafil, tadalafil, and vardenafil, were also found to downregulate the expression of both *Arg1* and *iNOS* by MDSCs, thereby inhibiting their suppressive function in growing tumors. This coincided with the enhancement of infiltration of T cells and the induction of a measurable anti-tumor immune response and a marked delay of tumor outgrowth in human cancer patients [49, 119]. ROS inhibitors including non-steroidal anti-inflammatory drugs (NSAID) like aspirin have also been shown to be effective for decreasing MDSC-mediated immune suppression in tumor-bearing mice [49]. Nitroaspirin, a compound coupled with a NO-releasing moiety and a conventional NSAID, has proven to be more efficient in means of inhibiting the production of ROS by limiting activities of *Arg1* and *iNOS*. Nitroaspirin inhibited the function of MDSCs and increased the number and function of tumor antigen-specific T cells when administered in conjunction with endogenous retroviral gp70 antigen [49, 120]. As a recent approach to target lipid metabolism of MDSCs, two FAO inhibitors, etomoxir and ranolazine, showed promising results that they inhibited FAO enzymatic pathway enzymes of MDSCs, CPT-1 and HADHA in a mouse model, respectively. Similarly, other FAO inhibitors including perhexiline (CPT1 inhibitor), trimetazidine (an HADHA inhibitor), or a lipase inhibitor such as orlistat were suggested to inhibit MDSC functions in the study, but their effects on tumor MDSC expansion and functions were not experimentally tested [93].

### Inhibition by other new applications

There are a broad range of new approaches including IL-12 immunotherapy and targeting of galectin-1, fibrinogen-like protein 2 (FGL2) and CD200 to control the number of MDSCs and functions. [22]. IL-12 has been known to promote lymphocyte proliferation, Th1 type polarization, and enhance IFNγ secretion of T and NK cells. Interestingly, recent IL-12 immunotherapy also demonstrated significant reduction of tumor-infiltrating MDSCs (50% decrease) and overexpression of CD80 and MHC II by MDSCs, suggesting to switch them towards an M1-type antigen-presenting cell phenotype and a potential therapeutic use [121]. Galectin-1 (Gal-1) is an immunosuppressive glycan-binding protein, which is up-regulated in several types of cancers including glioblastoma. Through interactions with β-galactoside-expressing glycoproteins on the T cell surface, Gal-1 can negatively regulate T cell survival and antagonize effector T cell signaling [122]. Silencing glioma-derived Gal-1 with a miRNA vector significantly prolonged the survival of glioma-bearing mice by decreasing the accumulation of glioma-infiltrating microglia/macrophages and MDSCs [22, 122].

Recent studies showed that FGL2 plays a role in MDSC glioma accumulation [123, 124]. FGL2 was shown to be upregulated in glioblastoma and correlates with glioma grade and tumor growth. It induces *CD39* gene expression in glioma-associated lymphocytes such as T cells. CD39 converts ATP to adenosine, which then suppresses T cell effector function, but promotes the tumor supportive role of MDSCs. The reduction of CD39 activity is also associated with diminished M2-type macrophage and MDSC accumulation in glioma. The use of an anti-FGL2 antibody in GL261-bearing mice reduced not only the number of MDSCs, but also the numbers of Treg and TAMs. A humanized anti-FGL2 antibody is currently in the process of being developed [22].

**Figure 3:**
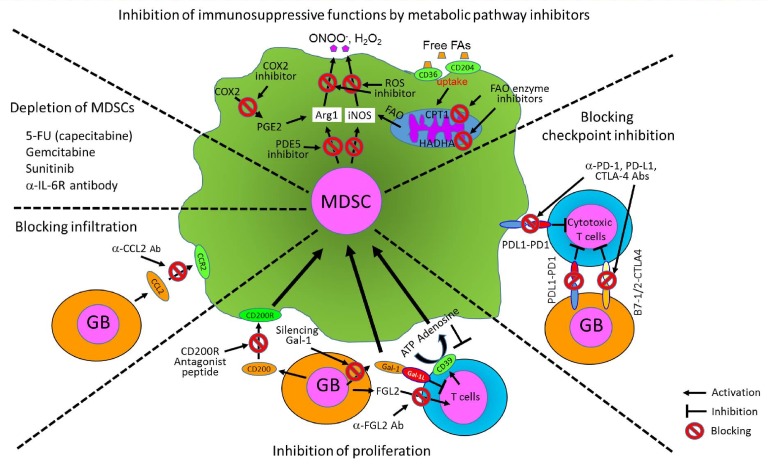
FIGURE 3: Therapeutic approaches to block the growth and immunosuppressive functions of MDSCs for glioblastoma treatment. The survival and immune functions of MDSCs can be controlled by various strategies such as targeting metabolic pathways, checkpoint inhibition, depletion of MDSCs, or blocking other functions such as proliferation and migration of MDSCs into the TME in several mouse glioma models and clinical trial studies for human glioblastoma patients.

Immunosuppression in gliomas is also regulated by interaction between tumor-derived CD200 and its receptor (CD200R) on myeloid cells. A CD200R antagonist peptide (A26059) has been shown to block the expansion of MDSCs, reduce secretion of Arg1 of MDSCs, and activate the CD8^+^ T cell response [125]. Here, we summarize various therapeutic strategies to control MDSCs for glioblastoma treatment from current literature in **Fig. 3**. These targeting approaches have been tested and are currently being tested in mouse glioma models and multiple clinical trials for human glioblastoma patients.

## IMMUNOTHERAPEUTIC APPLICATIONS TO CONTROL GLIOBLASTOMA

### Conventional standard of care treatment and immunotheraphy; complication and consideration of TMZ chemotherapy with immunotherapy

Following surgical removal of the tumor, glioblastoma patients are exposed to an aggressive treatment regimen that consists of concomitant fractionated radiotherapy with TMZ chemotherapy, followed by adjuvant TMZ. Although TMZ selectively targets fast-proliferating tumors by alkylating DNA at an early time point, but chemoresistance quickly follows and recurrent glioblastoma becomes the most challenging tumor with no alternative treatment. Immunotherapy has been recognized as a good alternative strategy to treat these recurrent chemoresistant gliomas. In addition, TMZ also has immunosuppressive side effects when administered systemically and thus can represent a major challenge for effective anti-cancer immunotherapy-based strategies by affecting the immune system [4]. Fadul *et al*. showed that the percentages and absolute cell numbers of NK cells among peripheral blood mononuclear cells isolated from radiotherapy and TMZ-treated glioblastoma patients were reduced [126].

On the other hand, some studies showed that TMZ chemotherapy can give beneficial effects on anti-tumor responses in glioblastoma. Curtin *et al*. demonstrated that radiation/TMZ-treated glioma cells undergo apoptosis and release the high mobility group box 1 protein (HMGB1), a Toll-like receptor 2 agonist, that acts on DCs to cause their activation and stimulates tumor antigen-specific T cell clonal expansion for anti-glioblastoma immune response [1, 4]. Zhang *et al*. showed that TMZ can suppress invasiveness of GSCs by down-regulating TGFβ2, giving a beneficial effect on glioblastoma control [127]. Thus, because the exact effect of TMZ to the immune system is not fully understood, it will need thorough understanding and investigation of TMZ action on the immune system and other normal cells, when combining TMZ chemotherapy with other immunotherapy-based strategies.

### Ongoing immunotherapy and combined approaches for glioblastoma treatment

Anti-cancer immunotherapy started various strategies that are intended to stimulate the patient's immune system against her/his own cancer and to promote immune-mediated anti-tumor responses [4]. However, the recent recognition that immune suppression has a crucial role in promoting tumor progression and frequent failures of cancer vaccines to induce an immune response has resulted in a paradigm shift regarding approaches for cancer immunotherapy [22, 49]. Despite the long history of glioblastoma treatment, current standard of care with TMZ-chemotherapy has shown only minor improvement. Thus, immunotherapy has become a promising and attractive alternative to treat glioblastoma, compared to non-specific use of cytotoxic TMZ-chemotherapy treatment modalities for several reasons.

First, it has great advantage to harness the body's own immune defenses to only attack against abnormal tumors and immune surveillance mechanism can detect appearance of abnormal tumors and keep it under control before starting expansion. Ultimately, the eradication of tumors is dependent on the activity of the adaptive immune system to recognize tumor neoantigens, overcome the immunosuppressive nature of the TME, and mount an effective immune response against the tumor. Secondly, given the uniqueness of the brain in its inaccessibility and diffusive growth nature of glioblastoma it is very challenging to resect glioblastoma precisely by neurosurgeons. However, immunotherapy allows anti-tumor T, NK cells, or glioma-specific engineered viruses to reach and only target and inhibit glioma at the single cell level. Finally, owing to recent technological breakthroughs, various new immunotherapy approaches have been developed over the years. Most of these approaches were originally developed for treatment of cancers other than brain cancers and they can be tested for glioblastoma treatment in various immunotherapy clinical trials [4].

Some of the new cellular targeting approaches such as galectin-1 knock-down, IL-12 administration, anti-CCR2 or anti-FGL2 might be effective and beneficial for glioblastoma therapy because these can reduce numbers of both MDSCs and GAMs. These therapeutic approaches were proven to work well in GL261 mouse models, but are not tested yet in a clinical trial setting [22]. Among ongoing clinical trials for glioblastoma, phase I clinical trial studying the effects of anti-PD-1 and anti-CTLA-4 combination therapy with angiogenesis-blocking antibody (bevacizumab) for recurrent glioblastoma (NCT02017717) and a number of studies of DC vaccines, peptide vaccine, CD133-targeting, and vaccine with glioma-associated antigens in recurrent and newly diagnosed glioblastoma are in progress (NCT02010606, NCT02149225, NCT02049489, NCT01808820, NCT02078648). These trials are being expected soon to give very promising and improved results [13]. Other ongoing immunotherapeutic approaches for glioblastoma clinical trials, antibody-drug conjugates (ADC), MDSC and GAM-targeting therapy, anti-angiogenesis therapy, C-X-C chemokine receptor type 4 (CXCR4 or CD182) inhibitor, oncolytic viral therapy, CAR T cell therapy, immune-stimulators, receptor tyrosine kinase (RTK) inhibitors or in combination are also currently being tested extensively in multiple clinical trials [1, 3, 4].

### Metabolic inhibition of MDSCs as a new target for drug development

Tumor environmental factors such as cytokines, chemokines, growth factors, or metabolites, initiate and direct metabolic reprogramming of MDSCs and lead to immunosuppression in various tumor models [83, 87, 90]. However, detailed mechanisms underlying these by which the specific factors lead to metabolic changes in glioblastoma remain largely unknown [52]. Fortunately, several recent studies with solid tumor models have shown that the functions of MDSCs are regulated by FAO through the PPARγ and mTOR pathway [94, 128]. These results strongly suggest that specific targeting metabolic pathways of MDSCs such as lipid metabolic pathway and mTOR pathway without affecting other cells would be a new promising therapeutic approach to control solid tumor as well as glioblastoma progression.

These approaches can be used as a combination therapy with TMZ chemotherapy, other immunotherapies, or current metabolic inhibitor drugs for glioblastoma treatment. Experimental examination with commercially available inhibitors of the LAL or mTOR pathway has currently not been studied for inhibiting growth and immunosuppressive functions of MDSCs in glioblastoma. Those inhibitors can be tested if they may block the glycolysis pathway or the FAO pathway of MDSCs and thus inhibit their expansion or immune functions in pre-clinical mouse models. Because both MDSCs and glioma (or GSCs) prefer the OXPHOS pathway over glycolysis it would be particularly interesting to examine whether FAO inhibitors can control the expansion of both glioma and MDSC in glioblastoma pathogenesis. In addition, GEM mouse models with deficiencies in the FAO, LAL or mTOR pathway would establish valuable models to investigate the *in vivo* metabolic and pathological roles of each component of these pathways in glioblastoma.

### Recent improvement of mouse glioblastoma xenograft models for pre-clinical drug study

A lot of progresses to date have been made in a syngeneic (C57BL/6) and orthotopic xenograft mouse model with GL261 mouse glioma cell line for glioblastoma studies [129-131], but this xenograft mouse model has several shortcomings [132]. First, cell lines cannot mimic *in vivo* patient glioblastoma tumor cells. Second, mouse and human have many differences in immune systems, such as biological factors with homologous, but different amino acid sequences and 3D structures, different immune cell composition, and distinct anatomic and physiological structures. For these reasons it is difficult to directly translate traditional glioblastoma xenograft mouse models to human glioblastoma cases and test drug candidates with these mouse models for pre-clinical study [133, 134]. Recently, NSG (nod, scid, gamma) mouse models, which are under NOD background, scid, and common gamma chain deficient, are being evaluated with patient-derived xenograft (PDX) samples and more sophisticated designed GEM mouse models have been developed [132]. These technological advancements in mouse model systems will enable the establishment of new whole-organismal systems that can mimic the human immune system more closely in response to various human cancers, including glioblastoma in mice, and can thus be used for testing candidate drugs more precisely for pre-clinical or co-clinical studies. Along with new or FDA approved therapies, previously developed drug portfolio, or combined approaches, these new humanized NSG mouse xenograft models would overcome mouse-human translation problems and provide a more reliable and closer platform to human for testing therapeutic efficacies for preclinical setups and speed up the drug development process. The new application of advanced mouse models into glioblastoma study would also give useful and critical information for improvement of glioblastoma patient treatment.

## FUTURE DIRECTIONS

To date, studies on immunosuppressive and pro-tumor functions in glioblastoma have heavily focused on GAMMs with preferential M2-type macrophage functions [1, 31, 32]. Although current information about functional roles of MDSCs in immunosuppression in glioblastoma is limited, evidence is accumulating that MDSCs are also another important immunosuppressive driver of glioblastoma pathogenesis [1, 13, 22]. Brain oncologists will need to broaden their research interests into MDSCs, elucidate mechanisms of MDSCs in glioblastoma models and target immunosuppressive and pro-tumoral functions of MDSCs for glioblastoma treatment. Altogether, control of MDSCs is a promising cellular therapeutic target for glioblastoma treatment.

## Abbreviations:

ATRA: all-trans retinoic acid,
BBB: blood brain barrier,
BM: bone marrow,
CCL: chemokine (C-Cmotif) ligand,
CCR: chemokine (C-Cmotif) receptor,
CNS: central nervous system,
COX: cyclooxygenase,
DC: dendritic cell,
e-MDSC: early stage MDSC,
FA: fatty acid,
FAO: FA oxidation,
GAMM: glioma-associated microglia/macrophages,
GB: glioblastoma,
GBM: glioblastoma multiforme,
GEM: genetically modified,
GSC: glioma stem cell,
iNOS: inducible nitric oxide synthase,
KIR: killer cell immunoglobulin-like receptor,
LAL: lysosomal acid lipase,
MDSC: myeloid-derived suppressor cell,
MHC: major histocompatibility complex,
MIF: macrophage migration inhibitory factor,
MMP: matrix metalloproteinase,
mTOR: mechanistic target of rapamycin,
NK: natural killer,
NSG: nod, scid, gamma,
OXPHOS: oxidative phosphorylation,
PMN: polymorphonuclear,
RNS: reactive nitrogen species,
ROS: reactive oxygen species,
SCF: stem cell factor,
TAM: tumor associated macrophage,
Tc: cytotoxic T cell,
TCA: tricarboxylic acid,
TCR: T cell receptor,
TME: tumor microenvironment,
Treg: regulatory T cell,
TMZ: temozolomide,
VEGF: vascular endothelial growth factors,
WHO: world health organization.
